# Retraction: Establishment of a non-small-cell lung cancer-liver metastasis patient-derived tumor xenograft model for the evaluation of patient-tailored chemotherapy

**DOI:** 10.1042/BSR20182082_RET

**Published:** 2026-05-06

**Authors:** Xue Yang, Gaopei Meng

**Affiliations:** Cangzhou Central Hospital, No. 16 Xinhua West Road, Cangzhou 061000, Hebei, China

**Keywords:** DOX, DTX, Metastasis, NSCLC, PDTX

This article is being retracted from *Bioscience Reports* at the request of the Editor-in-Chief and the Editorial Board. This follows the receipt of a notification from a reader, alerting the Editorial Board to multiples similarities detected between this manuscript and those published by other authors.

Specifically, these similarities include:

- Similarities between the Figure 2A F0 and F2 panels and the Figure 1A HE staining panels a and b of Lu et al. (2015) (DOI: 10.18632/oncotarget.4729) following 180-degree rotation
- Similarities between the Figure 2A F1 panel and the Figure 1A HE staining panel c of Lu et al. (2015)
- Similarities between the Figure 2B F0 panel and the Figure 2G Adenocarcinoma TNF-α panel of Lu et al. (2015)
- Similarities between the Figure 2B F1 and F2 panels and the Figure 2G Normal and Squamous carcinoma TNF-α panels of Lu et al. (2015) following 180-degree rotation
- Similarities between the Figure 3A F1 panel and Figure 3A of Zhuang et al. (2017) (DOI: 10.1590/1414-431X20176000)
- Similarities between the Figure 3A F2 panel and Figure 3B of Zhuang et al. (2017) following 180-degree rotation

The authors have been contacted with regard to the retraction and have not responded to the Journal's queries or the concerns raised. Given the extent of the issues raised, the Editorial Board stands by the decision to retract the article.

